# Immobilization of Endonuclease A from *Serratia marcescens* on cellulose membranes

**DOI:** 10.1007/s10529-026-03728-2

**Published:** 2026-06-03

**Authors:** Tobias Schneider, Michael Wolff

**Affiliations:** Technische Hochschule Mittelhessen, Wiesenstraße 14, 35390 Gießen, Germany

**Keywords:** Cellulose, Endonuclease A, Enzyme immobilization, Enzyme technology

## Abstract

The removal of ds-DNA constitutes an indispensable step in numerous biotechnological production processes. In many cases, endonucleases are employed for this purpose. The objective of this study was to investigate the immobilization of Endonuclease A from *Serratia marcescens* onto cellulose filters, which could later be used in biotechnological production processes. The filters were treated with *p*-toluenesulfonyl chloride prior to enzyme functionalization, and tosyl loading was confirmed by HPLC. Enzyme activity after immobilization was investigated using a colorimetric assay. The functionalization of the filters was most effective when they were treated with 0.5 M NaOH prior to reaction with *p*-toluenesulfonyl chloride. The subsequent endonuclease immobilization process was screened under various pH conditions and was most successful when conducted at a slightly acidic pH. However, it was also observed that functionalization with *p*-toluenesulfonyl chloride was not necessary to immobilize the endonuclease onto the cellulose membranes, as initially assumed. This indicates that immobilization can proceed through a pathway independent of the presence of tosylate groups on the surface of the cellulose filters.

## Introduction

The immobilization of enzymes in solid substrates has been the subject of academic and commercial interest for several decades. The recovery of an immobilized enzyme is a more straightforward process, and the separation of catalyst and product is greatly facilitated. In addition, immobilized enzymes often demonstrate enhanced stability and activity retention compared to their free forms (Homaei et al. 2013). Consequently, an enzymatic industrial process is often optimized by the utilization of immobilized enzymes. There are several methods employed to immobilize enzymes on a solid substrate, including adsorption, covalent binding, affinity immobilization, and entrapment (Datta et al. 2013).

Endonucleases are a class of enzymes that are frequently used in industrial contexts for the purpose of removing unwanted polynucleotides from cell lysates. A notable member of this group is Endonuclease A from *Serratia marcescens*, which is utilized in biotechnological processes. It is a homodimer with a mass of 57.8 kDa that exhibits a slight preference for G-C-rich segments and cleaves DNA and RNA into 2–5 nucleotide segments (Nestle and Roberts 1969a; Moreno et al. 1991a). The enzyme was reported to show maximal processivity at a pH of 8 and 37 °C and is used generally within this range (Chen et al. 2011). While pH 8 is optimal for the enzyme, it was shown to be active between pH 5–11, while heating above 44 °C inactivates the enzyme (Nestle and Roberts 1969b). Molecular dynamics studies showed that the monomeric form of Endonuclease A exhibits some DNase activity as well, however the dimeric form is more active (Chen et al. 2006, 2007). Endonuclease A has also been commercialized in various forms (Benzonase, DNARASE, Turbo Nuclease).

In this study, we investigated the immobilization of Endonuclease A onto cellulose, as cellulose is a readily available and inexpensive material that is used in many technical applications.

Previous studies have investigated the immobilization of this enzyme. As early as 1991, the immobilization of Endonuclease A was reported in corn cob, which has a similar composition to wood, consisting primarily of cellulose, hemicellulose and lignin (Moreno et al. 1991a; 1991b). *p*-Toluenesulfonyl chloride can be used as an activating agent for a cellulose substrate, on which the enzyme can be covalently immobilized in a second step. Other reports have employed alternative immobilization reagents, such as glutaraldehyde for covalent immobilization (Yin et al. 2018).

In the present study, we expand the toolbox for immobilization and activity assessment of Endonuclease A on cellulose membranes.

## Materials and methods

### Chemicals and filters

Endonuclease A was acquired from c-Lecta GmbH, Leipzig, Germany (DNARASE®, 250 U/µL). Enzyme concentration was estimated to be 5 mg/mL using a colorimetric BCA assay, which was previously described (Lothert et al. 2020). *p*-Toluenesulfonyl chloride (99% purity) was purchased from TCI chemicals (Tokyo, Japan). Dried acetone was obtained from Thermo-Fisher (Waltham, USA). Pyridine (> 99.8% purity, dry) was purchased from Sigma Aldrich (St. Louis, USA). The filters Whatman® RC60 composed of regenerated cellulose, were purchased from Merck (Darmstadt, Germany). The Quant-iT™ PicoGreen™ ds-DNA Assay was purchased from Thermo Fisher (Waltham, USA).

## Mercerization

The cellulose filters were submerged for a period of 4 h in a solution of 0.5 M NaOH, after which they were washed with 20 mL of water (3 ×) in order to remove any residual NaOH. After the third wash, neutral conditions were confirmed in the aqueous solution using pH indicator paper. Subsequently, the filters were washed with acetone (2 ×) and dried for 1 h with compressed air to remove excess solvent before further use.

## Tosylation

The mercerized and dried filters were soaked in 4 mL of pyridine for a period of three hours. Thereafter, freshly prepared 0.2 M *p*-toluenesulfonyl chloride in acetone was added (5 mL per mL of pyridine). The mixture was then stirred gently for a period of 18 h. Then, the filters were washed with 20 mL of acetone (3 ×), 20 mL of 5 mM HCl (2 ×), and 20 mL of deionized water (2 ×).

## Immobilization of Endonuclease A

In order to facilitate immobilization, the filters were cut into smaller fragments. One-eighth of a filter was placed into 25 mL sample tubes containing 1 mL buffer (50 mM citrate buffer for pH 3, 4, 5 and 6, 50 mM carbonate buffer for pH 8 and 10). The buffer completely submerged the filter and 1 µL of enzyme was added. The filter was incubated at 4 °C for 24 h without agitation. Upon completion, the incubation solution was discarded from the sample tubes and 5 mL of 10 mM KCl solution was added. The tubes containing 10 mM KCl and the filters were then gently shaken at 6 °C for 15 min, and the washing solution was discarded afterwards. This process was repeated four more times in order to remove any non-immobilized enzyme and residual buffer from the filters. The last washing solution was then tested for residual DNase activity using the PicoGreen assay described below in order to exclude the presence of non-immobilized enzyme in the filters.

## Determination of tosylate group activation of cellulose filter

In order to quantify the amount of tosylate groups, activated filters were treated with 1 M NaOH. The samples from the supernatant were analyzed using an Ultimate 3000 HPLC system (Thermo Fisher), equipped with Ascentis® C-18 column (25 cm × 4.6 mm) by a RS Diode Array detector. The samples were collected from the supernatant until the tosylate concentration was stable. The concentration of tosylate in the sample was then determined (10 µL injection volume, 0.1% formic acid in water (A) and 0.1% formic acid in acetonitrile (B), 0–15 min; 5%−100% B; 1 mL/min, at RT), and this value was used to calculate the total amount of tosylate that had been liberated from the filters. The wavelength used for measurements was 260 nm.

## Immobilized endonuclease activity assay

To evaluate the filter activity, a modified Quant-iT™ PicoGreen™ assay was employed to quantify ds-DNA (λ-DNA standard from PicoGreen kit) concentration in the wells. The assay was modified the following way: Component B (incubation buffer) of the assay contains 1 µM EDTA and 10 µM Tris–HCl at pH 7.5. This was replaced by a solution of 2 mM Mg and 10 mM Tris–HCl at pH 8 in order to prevent inhibition of the immobilized enzyme. To each well in a 96 well plate suitable for fluorescence measurements was added 100 µL of 2 µg/mL ds-DNA stock (200 ng ds-DNA per well) and 100 µL of diluted Quant-iT™ PicoGreen™ reagent (5 µL concentrated reagent in 2 mM Mg and 10 mM Tris–HCl at pH 8). Afterwards, the fluorescence baseline level was determined. The filter fragments were cut into 6 mm diameter discs using a stapler. The 6 mm filter discs were then placed into the wells containing ds-DNA and the PicoGreen™ reagent and incubated at room temperature in darkness. Prior to each measurement, the filters were removed from the wells, as their presence heavily impeded the detection of the fluorescence signal. Immediately upon completion of the measurement, the filters were placed back inside their respective wells for continued incubation. Data were collected using a BioTek Cytation3 plate reader (Agilent). All measurements were performed in triplicate independent experiments (n = 3).

## Results

### Tosylation of filters

The tosylation was examined in both mercerized and unmercerized filters under a variety of pre-treatments. In our hands, the highest loading was achieved using a combination of mercerization and pyridine pre-treatment. Table 1 shows the results of the tosylate loading experiments.

**Table 1:** Conditions and achieved tosylate loading of cellulose filters (in mg tosylate per g cellulose)

Mercer-ization	Pre treatment	Tosylation	Achieved loading of tosylate
+	Pyridine 18 h	0.2 M TsCl in acetone (18 h)	3,4 mg/g
–	Pyridine 18 h	0.2 M TsCl in acetone (18 h)	2,3 mg/g
+	Pyridine 1 h	0.2 M TsCl in acetone (18 h)	3,2 mg/g
-	Pyridine 1 h	0.2 M TsCl in acetone (18 h)	2,0 mg/g
+	none	0.2 M TsCl + 0.4 M Pyridine in acetone (18 h)	2,0 mg/g
–	none	0.2 M TsCl + 0.4 M Pyridine in acetone (18 h)	2,0 mg/g

Mercerization and pretreatment with pyridine increased the amount of tosylate groups which were covalently bound to the surface of the filters. When combined, mercerization and pyridine pre-treatment produced the filters carrying the highest amount of tosylate groups. A prolonged treatment time with pyridine treatment had an additional, but smaller effect. Therefore, only mercerized filters treated with pyridine for 18 h were used in all subsequent experiments.

### Immobilization of Endonuclease A

**Fig. 1:**
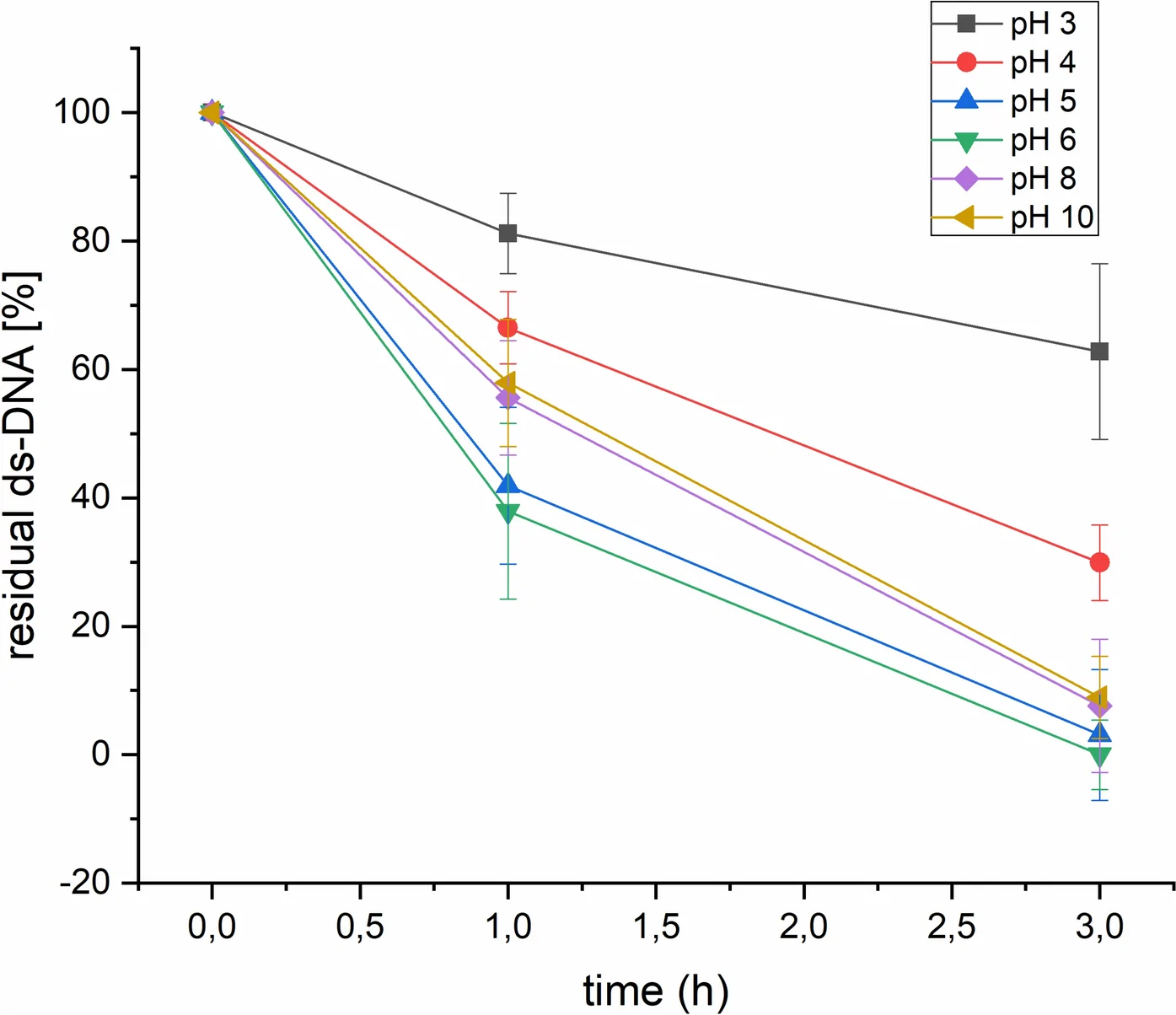
Activity of filters depending on immobilization pH using tosylated filters with standard error of the mean (*n* = 3)

When tosylated filters were treated in a solution of 10 mM buffers with pH values between 3 and 10, a pronounced activity of the resulting filters could be determined after repeated washing of the filters with 10 mM KCl solution (Fig. 1). Filters treated with enzymes in buffers at pH 5 and pH 6 degraded more ds-DNA compared to filters incubated under different conditions. Treatment in more basic media was slightly less effective, but still superior to treatment in acidic buffers with pH ≤ 4.

**Fig. 2:**
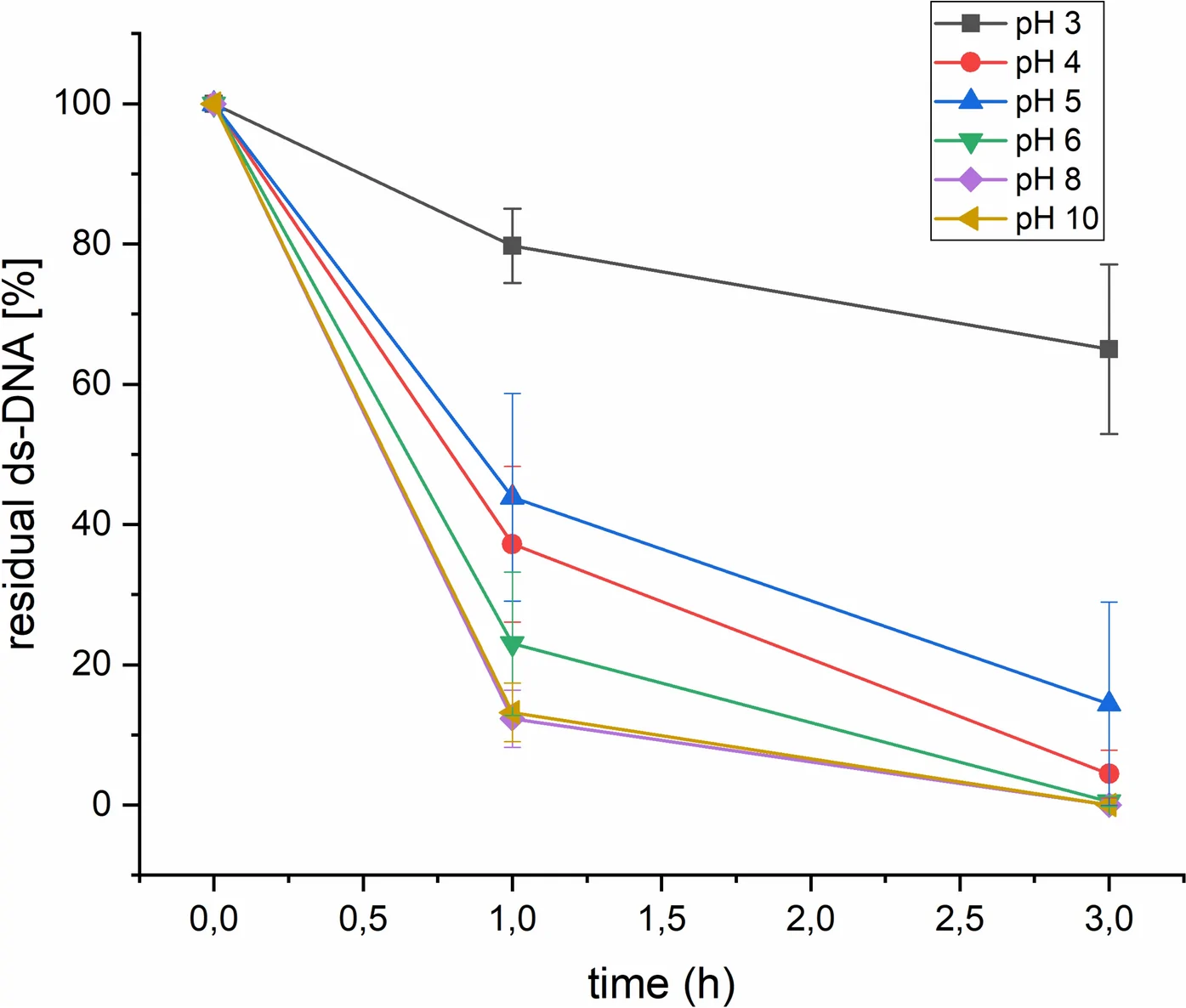
Activity of supernatant solution depending on immobilization pH using tosylated filters with standard error of the mean (*n* = 3)

To differentiate between a loss of enzyme function resulting from treatment conditions and unsuccessful immobilization, we conducted assays of enzymatic activity utilizing the supernatant solution employed for immobilization (Fig. 2). Here, we found that supernatants from acidic buffers did also show a comparatively low ability to degrade ds-DNA, while increasing pH led to an increased activity of the filters.

Filters incubated with enzyme in a buffer with a pH of 2 did not exhibit any capacity to degrade ds-DNA, as did their respective supernatants (data not shown).

**Fig. 3:**
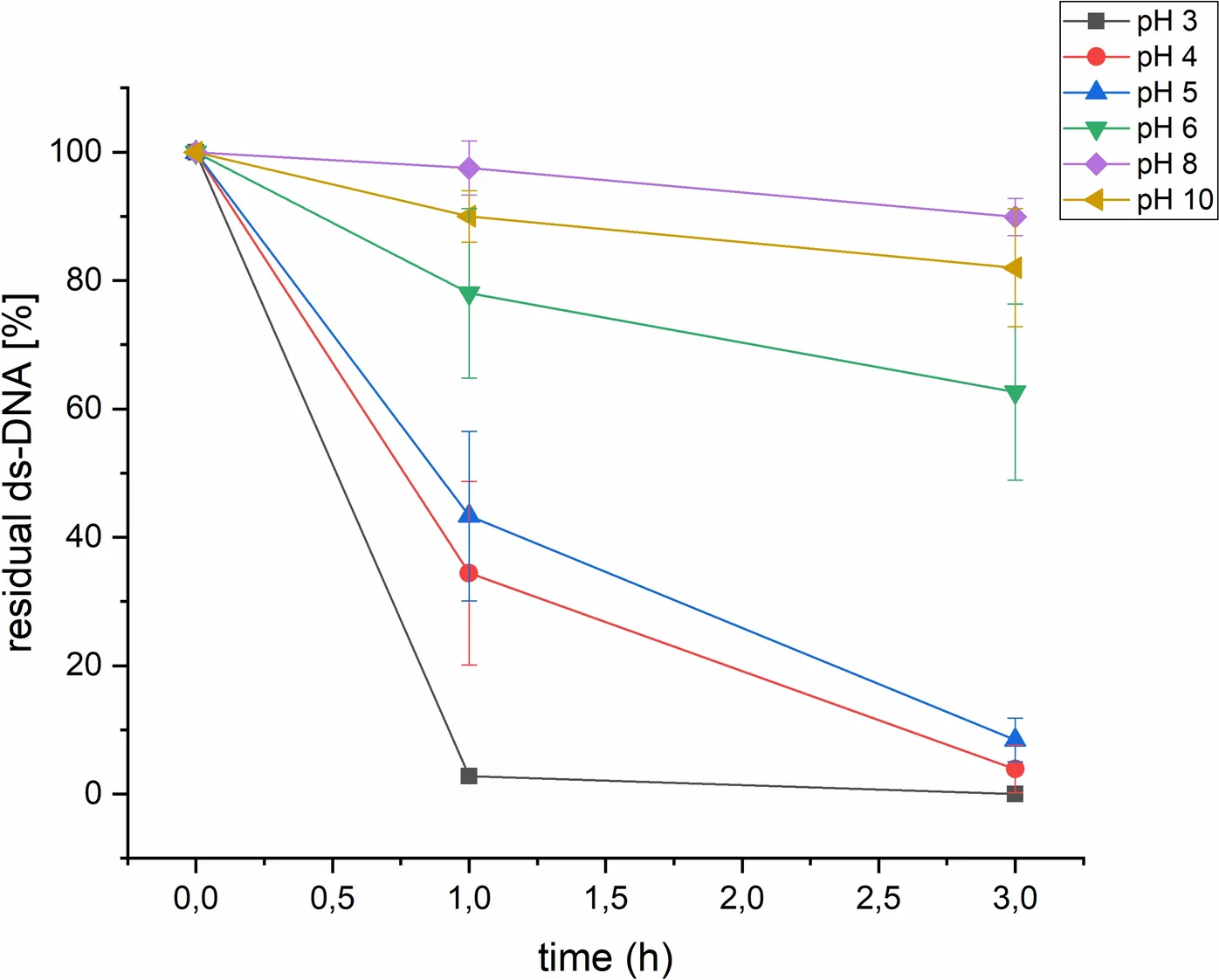
Activity of filters depending on immobilization pH using non-tosylated filters with standard error of the mean (*n* = 3)

In order to complement the experiments with filters which underwent tosylation, we also sought to investigate whether tosylation is ultimately responsible for the immobilization of the enzyme onto the membranes (Fig. 3). Consequently, we conducted experiments with non-tosylated membranes, which otherwise received the same treatment as the tosylated membranes. Our findings demonstrated that immobilization did occur under acidic conditions and appeared to become less effective when the pH is raised from 3 to 8.

**Fig. 4:**
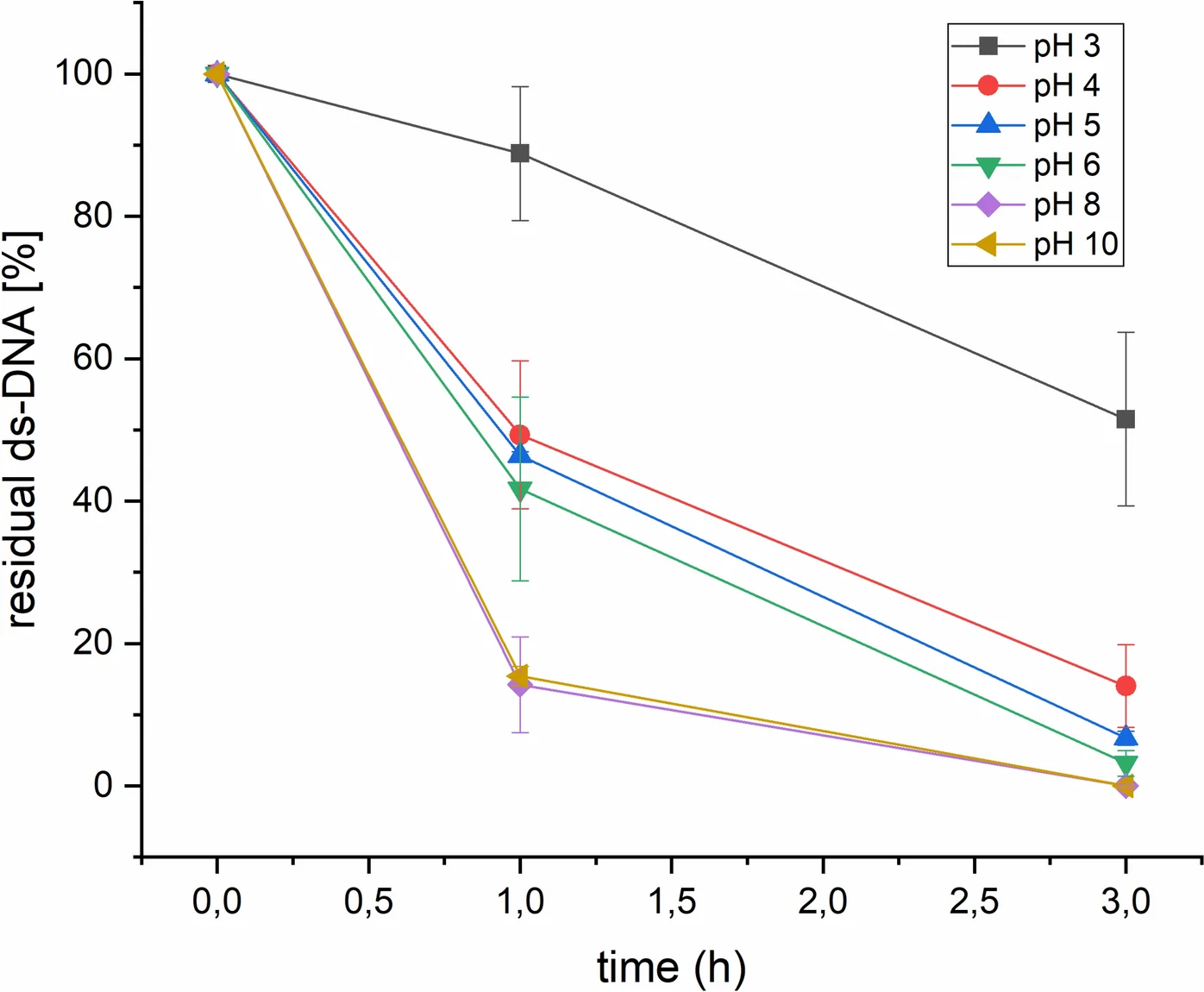
Activity of supernatant depending on immobilization pH using non-tosylated filters with standard error of the mean (*n* = 3)

The respective supernatants of the non-tosylated filters followed the inverse relationship: the supernatant of immobilization at pH 10 was most active, while residual ds-DNA concentration increased with more acidic pH values (Fig. 4).

To ascertain whether external factors were responsible, the assay was performed with identical conditions as above, except that immobilization was carried out without the addition of endonuclease. No degradation of ds-DNA was observed under any pH condition.

### Stability of immobilized enzyme

A significant challenge impeding the renewed use of enzymes is their inherent tendency to diminish in activity over time. Our research also sought to ascertain whether the free or the immobilized form of Endonuclease A exhibited a greater proclivity towards reduced activity.

Our findings indicate a notable dependency of the enzyme in question on storage temperature. A storage at 37 °C resulted in a complete loss of the ability to break down ds-DNA after only 18 h. In contrast, filters stored at 6 °C demonstrated noticeable activity after one week, although the rate of DNA degradation was reduced to approximately one-third of that observed in freshly prepared filters. Enzyme immobilized on filters and free enzymes exhibit comparable abilities to break down ds-DNA relative to the freshly prepared state.

## Discussion

### Tosylation of filters

The tosylate loading experiments demonstrated a discernible tendency towards elevated loadings when mercerization preceded the treatment with pyridine and *p*-toluenesulfonyl chloride (TsCl). This finding is consistent with previously reported data (Albayrak and Yang 2002), which indicated that mercerization leads to a swelling of cellulose fibers, thereby increasing the number of accessible hydroxyl groups for subsequent functionalization with TsCl. Furthermore, the addition of pyridine to the treatment process has been shown to enhance the loading efficiency when combined with mercerized filters. This improvement may be attributed to the ability of cellulose to break intermolecular hydrogen bonds, thereby increasing the accessibility of the cellulose fibers for reaction with TsCl (Labafzadeh et al. 2012).

### Filter activity

One goal of this study was to screen for optimal immobilization conditions. In this context it was investigated how the buffer pH affects the effectiveness of enzyme immobilization. Previous experiments have used pH 8 for immobilization, as this is the pH at which Endonuclease A has its maximum activity (Moreno et al. 1991b). However, optimal conditions for immobilization and enzymatic activity do not necessarily need to be identical.

The immobilization of Endonuclease A on activated cellulose filters revealed that the filters exhibited the greatest enzyme activity when immobilized at slightly acidic pH (5–6) compared to all other tested pH conditions. The immobilization was less effective at the pH of the enzymatic activity optimum (pH 8) or above. The supernatant solutions demonstrated an inverse trend, indicating a lower degree of immobilization at higher pH, while the endonuclease remains active in solution. However, at acidic pH with tosylated filters, both the filters and the supernatant exhibit a diminished capacity to degrade ds-DNA. This observation suggests that the enzyme in question is prone to inactivate at pH values below 4. Consistent with this assumption is our finding that no ability to break down ds-DNA, neither in filters or in supernatant solution, was found when a pH 2 buffer was used for enzyme immobilization.

Furthermore, the immobilization of enzymes to the filters at higher pH levels appears to be an ineffective approach, as evidenced by the observation that the supernatant solution continues to exhibit clear enzymatic activity at pH values of 8 and 10. This is unexpected as it is generally assumed that the lysine ε-NH_2_ groups of the enzyme are covalently attached to the displaced tosyl group of the activated cellulose substrate via nucleophilic substitution reactions. However, the ε-NH_2_ groups of lysine have a reported pKa value of 10.28, meaning that below a pH value of 8, the non-nucleophilic ammonium form of the lysine side chain should predominate. It is therefore likely that other effects play an important role in the immobilization of Endonuclease A on tosylated cellulose supports. To investigate the exact role of the tosyl groups, we repeated the experiments with filters that were not treated with TsCl, but otherwise analogous to the filters used previously.

Surprisingly, these filters also showed clear evidence of enzyme immobilization. Filters incubated at pH ≥ 6 showed little activity, whereas activity increased significantly with decreasing pH. The supernatant showed the opposite trend, indicating effective immobilization at acidic pH and removal of the target enzyme from the supernatant. This form of immobilization is clearly not dependent on the presence of tosylate groups and preferentially occurs at acidic pH. However, it is not clear whether covalent bonds are formed or whether this immobilization is mediated by weaker intermolecular forces. It appears that this immobilization form also benefits greatly from low pH-values, as it is able to offset any occurring enzyme denaturation at this pH value.

We also excluded the possibility that filters not treated with enzyme may have given false positive results in the assay used. Regardless of the treatment pH, all tosylated and non-tosylated filters showed no ability to degrade any ds-DNA when endonuclease treatment was omitted in our protocol.

### Stability of immobilized enzyme

The activity of the enzyme decreases with time during storage. At the activity optimum of 37 °C, the activity decreased rather rapidly, whereas storage at lower temperatures significantly increased the shelf life of both immobilized and free enzyme. This reduction in activity may be due to misfolding and denaturation of the enzyme structure or proteolytic degradation.

## Conclusion

We investigated the immobilization of Endonuclease A on regenerated cellulose filters. Activation of the cellulose support with *p*-toluenesulfonyl chloride was successful and was increased by pretreatment with NaOH and pyridine. We found the immobilization of the enzyme was highly dependent on the pH value. However, filters not treated with *p*-toluenesulfonyl chloride were also suitable substrates for immobilization, especially at acidic pH values. This observation suggests that the immobilization of Endonuclease A on cellulose does not require tosylate pre-treatment and is mediated by a different mechanism. More experiments are needed to confirm the immobilization mechanism. Over time, the activity of the enzyme decreased in both immobilized and free forms. The rate of decline was dependent on storage temperature.

## Data Availability

The datasets generated during and/or analysed during the current study are available from the corresponding author on reasonable request.

## References

[CR1] Albayrak N, Yang S-T (2002) Immobilization of *Aspergillus oryzae* β-galactosidase on tosylated cotton cloth. Enzyme Microb Technol 31:371–383. 10.1016/S0141-0229(02)00115-1

[CR2] Chen C, Beck BW, Krause K, Pettitt BM (2006) Solvent participation in *Serratia marcescens* endonuclease complexes. Proteins 62:982–995. 10.1002/prot.20694 16355414

[CR3] Chen C, Beck BW, Krause K, Weksberg TE, Pettitt BM (2007) Effects of dimerization of *Serratia marcescens* endonuclease on water dynamics. Biopolymers 85:241–252. 10.1002/bip.20641 17133507 PMC2583238

[CR4] Chen P, Yang H, Li H, Yang L, Li X (2011) Expression, purification and characterization of non-specific *Serratia* nuclease in *Escherichia coli*. Sheng Wu Gong Cheng Xue Bao 27:1247–1257 22097815

[CR5] Datta S, Christena LR, Rajaram YRS (2013) Enzyme immobilization: an overview on techniques and support materials. 3 Biotech 3:1–9. 10.1007/s13205-012-0071-7 28324347 PMC3563746

[CR6] Homaei AA, Sariri R, Vianello F, Stevanato R (2013) Enzyme immobilization: an update. J Chem Biol 6:185–205. 10.1007/s12154-013-0102-9 24432134 PMC3787205

[CR7] Labafzadeh SR, Kavakka JS, Sievänen K, Asikkala J, Kilpeläinen I (2012) Reactive dissolution of cellulose and pulp through acylation in pyridine. Cellulose 19:1295–1304. 10.1007/s10570-012-9720-6

[CR8] Lothert K, Offersgaard AF, Pihl AF, Mathiesen CK, Jensen TB, Alzua GP, Fahnøe U, Bukh J, Gottwein JM, Wolff MW (2020) Development of a downstream process for the production of an inactivated whole hepatitis C virus vaccine. Sci Rep 10:16261. 10.1038/s41598-020-72328-5 33004836 PMC7530675

[CR9] Moreno JM, Sanchez-Montero JM, Sinisterra JV, Nielsen LB (1991a) Contribution to the study of the enzymatic activity of benzonase. J Mol Catal 69:419–427. 10.1016/0304-5102(91)80120-R

[CR10] Moreno JM, Sanchez-Montero JM, Ballesteros A, Sinisterra JV (1991b) Hydrolysis of nucleic acids in single-cell protein concentrates using immobilized benzonase. Appl Biochem Biotechnol 31:43–51. 10.1007/BF02922124 1665680

[CR11] Nestle M, Roberts WK (1969) An extracellular nuclease from *Serratia marcescens*. J Biol Chem 244:5219–5225. 10.1016/S0021-9258(18)63649-X 4310088

[CR12] Yin J, Chen S, Zhang N, Wang H (2018) Multienzyme cascade bioreactor for a 10 min digestion of genomic DNA into single nucleosides and quantitative detection of structural DNA modifications in cellular genomic DNA. ACS Appl Mater Interfaces 10:21883–21890. 10.1021/acsami.8b05399 29882639

